# *Apiospora arundinis,* a panoply of carbohydrate-active enzymes and secondary metabolites

**DOI:** 10.1186/s43008-024-00141-0

**Published:** 2024-04-07

**Authors:** Trine Sørensen, Celine Petersen, Asmus T. Muurmann, Johan V. Christiansen, Mathias L. Brundtø, Christina K. Overgaard, Anders T. Boysen, Rasmus D. Wollenberg, Thomas O. Larsen, Jens L. Sørensen, Kåre L. Nielsen, Teis E. Sondergaard

**Affiliations:** 1 Department of Chemistry and Bioscience, Aalborg University, Fredrik Bajers Vej 7H, Aalborg, 9220 Denmark; 2 Department of Biotechnology and Biomedicine, Technical University of Denmark, Søltofts Plads 221, Kongens Lyngby, 2800 Denmark; 3 Department of Chemistry and Bioscience, Aalborg University, Niels-Bohrs Vej 8, Esbjerg, 6700 Denmark

**Keywords:** Oxford Nanopore sequencing, *Apiospora*, *Arthrinium*, CAZymes, Secondary metabolites

## Abstract

The *Apiospora* genus comprises filamentous fungi with promising potential, though its full capabilities remain undiscovered. In this study, we present the first genome assembly of an *Apiospora arundinis* isolate, demonstrating a highly complete and contiguous assembly estimated to 48.8 Mb, with an N99 of 3.0 Mb. Our analysis predicted a total of 15,725 genes, with functional annotations for 13,619 of them, revealing a fungus capable of producing very high amounts of carbohydrate-active enzymes (CAZymes) and secondary metabolites. Through transcriptomic analysis, we observed differential gene expression in response to varying growth media, with several genes related to carbohydrate metabolism showing significant upregulation when the fungus was cultivated on a hay-based medium. Finally, our metabolomic analysis unveiled a fungus capable of producing a diverse array of metabolites.

**Supplementary Information:**

The online version contains supplementary material available at 10.1186/s43008-024-00141-0.

## Introduction

Fungi have played a significant and enduring role in human history, providing us with numerous benefits for millennia. They have been integral to processes such as beer and wine production, cheese making, enzyme synthesis, and pharmaceuticals (Michel et al. 1992, 1993; Fleet 1999; Ropars et al. 2012; Hüttner et al. 2020; Meyer et al. 2020; El-Gendi et al. 2022). However, fungi also exert a negative impact on the global food chain by infecting crops and causing annual yield losses (Savary et al. 2019). To harness the untapped potential of fungi and address the challenge of pathogenic plant infections, a deeper understanding of these organisms is imperative. Recently, there has been a growing interest in leveraging fungi and their products to develop more sustainable solutions. In this quest for novel insights, it's crucial to broaden our exploration beyond extensively studied fungi like *Fusarium, Aspergillus,* and *Penicillium*. The genus *Apiospora*, in comparison, remains relatively underexplored, with only a limited number of genome assemblies and a sparse body of research on its transcriptome and metabolome (Mukherjee et al. 2016; Li et al. 2020; Eltivitasari et al. 2021; Fang et al. 2021; Heo et al. 2021; Majeedano et al. 2022; Petersen et al. 2022). Within the fungal kingdom, there lies a rich reservoir of bioactive secondary metabolites (Keller et al. 2005; Hoffmeister and Keller 2007), and the *Apiospora* genus is no exception, exhibiting a diverse array of secondary metabolites (Overgaard et al. 2022). Additionally, this genus has demonstrated a promising capacity to produce a multitude of carbohydrate-active enzymes (CAZymes) (Shrestha et al. 2015; Li et al. 2020; Majeedano et al. 2022). CAZymes, a class of enzymes capable of breaking down complex carbohydrates and glycoconjugates, enable fungi to degrade biomass and extract nutrients. Many of these enzymes are secreted from fungal hyphae and exhibit stability outside the cell, making them particularly valuable for environmentally friendly processes such as converting lignocellulose into biofuels and other valuable resources (Saini et al. 2015; Lange 2017; Meyer et al. 2020).

As for many other fungal genera, the size and members of the *Apiospora* genus remain dynamic (Wang et al. 2018; Kwon et al. 2021). Earlier, the *Apiospora* genus was phylogenetically placed in the same phylogenetic clade as *Arthrinium* (Crous and Groenewald 2013; Wang et al. 2018). Recently, studies have shown a phylogenetic delimitation of the *Apiospora* genus and the *Arthrinium* genus (Crous et al. 2021; Pintos and Alvarado 2021, 2022; Tian et al. 2021; Sørensen et al. 2022). The genus *Apiospora* comprises species that occur in a wide variety of geographic and ecological environments. The roles and habitats occupied by *Apiospora* include endophytes of plants (Agut and Calvo 2004; Ramos et al. 2010), lichen (He and Zhang 2012), algae (Suryanarayanan 2012), and cutaneous infections in humans (Rai 1989; Zhao et al. 1990; Greven et al. 2018). Infection of several different critical crops has also been reported for the *Apiospora* genus. One species belonging to the *Apiospora* genus that has been linked to infections in various crops is *Apiospora arundinis*. This association has led to significant crop losses annually (Martínez-Cano et al. 1992; Bagherabadi et al. 2014; Chen et al. 2014; Thangaraj et al. 2019; Liao et al. 2022). It is crucial to note that a potent neurotoxin, 3-nitropropionic acid, has been identified in several *Apiospora* species. This toxin has been responsible for numerous cases of poisoning and fatalities in China due to the consumption of moldy sugarcane (Wei et al. 1994) but single cases have also been found in Scandinavia, one of which was caused by an *Apiospora*-infected coconut (Birkelund et al. 2021; Bendiksen Skogvold et al. 2022).

This study aims to generate the first structurally annotated genome assembly of an *A. arundinis* species by employing long reads generated using Oxford Nanopore Technology (ONT), in conjunction with short reads produced using Illumina sequencing. The resulting genome assembly is then utilized to elucidate the genetic potential of the fungus and explore possible useful applications that the fungus might have. Furthermore, it aims to describe the genetic response of the fungus when cultivated on different media types. Lastly, we conducted a detailed investigation of the metabolic profile of the fungus cultivated on different media types, identifying several compounds produced by the fungus and their association with candidate gene clusters. This comprehensive analysis not only enhances our understanding of the fungal genome but also provides valuable insights into the potential applications and metabolic adaptability of *A. arundinis*, contributing significantly to the broader field of fungal genomics and applied biotechnology.

## Methods and materials

### Fungal isolation

The *Apiospora arundinis* isolate AAU 773 was isolated from the nest of a gull (*Larus argentatus)* in Aalborg, Denmark in 2016 and deposited in the fungal database at Aalborg University.

### Genomic DNA extraction and sequencing of long reads using Oxford Nanopore

The Genomic Buffer Set method described in Petersen et al. (2022) was used to extract high molecular weight (HMW) DNA from *A. arundinis* AAU 773 grown in liquid yeast extract sucrose (YES) media (150 g/L sucrose, 20 g/L yeast extract, 0.5 g/L Mg_S_O_4_ ⋅7H_2_O, 1 mL/L trace solution (16 g/L ZnSO_4_ ⋅7H_2_O, 5 g/L CuSO_4_ ⋅ 5H_2_O)) at 25 °C, for five days and at 150 rpm (Petersen et al. 2022). The mycelium was harvested according to the same protocol. The HMW DNA was subsequently purified, and small DNA fragments were removed according to the same protocol. Quality control of the extracted DNA was performed according to the same protocol. The HMW DNA was peppered for sequencing using the Ligation Sequencing Kit SQK-LSK109 (Oxford Nanopore Technologies, Oxford, UK) and the sequencing was performed on a FLO-MINI106D (R9.4.1) flow-cell (Oxford Nanopore Technologies, Oxford, UK). The raw reads were base called using Guppy version 3.6.1 in GPU mode using the dna_r9.4.1_450bps_hac.cfg model (Oxford Nanopore Technologies 2021).

### Genomic DNA extraction and sequencing of short reads using Illumina

Genomic DNA from ~25 mg freeze-dried mycelium grown in yeast extract peptone glucose (YPG) media (10 g/L yeast extract, 20 g/L peptone, 25 g/L glucose) was extracted with the FastDNA SPIN Kit for Soil (MP Biomedicals, USA) and subsequently cleaned with Agencourt AMPure XP beads (Beckman Coulter, USA) using a 1:0.7 (v/v) bead:sample ratio. A Nextera paired-end DNA library (Illumina, USA) was prepared according to the Nextera “DNA Library Prep Reference Guide” (Document # 15027987 v01), with the modification of using Agencourt AMPure XP beads (1:0.7 bead:sample ratio) for the post-tagmentation clean-up. DNA quality (A260/A280 and A260/A230), size distributions, and concentration were assessed on the NanoDrop ND-1000 (Thermo Scientific, USA), D1000 ScreenTape system (Agilent, USA) and using the Qubit dsDNA HS assay kit (Thermo Scientific, USA), respectively. The paired-end library was sequenced to approximately 900x coverage on the Illumina HiSeq 2500 system using the Rapid SBS Kit v2 (2x250 cycles).

### Genome assembly

The long reads were trimmed using a minimum quality of 80 using Filtlong version 0.2.0 (Wick 2018). Reads shorter than 20 kbp after filtering were discarded. Minimap2 version 2.17 (Li 2018) was used to create overlaps between the long reads, and Miniasm version 0.3 (Li 2016) was used to create the genome assembly. The raw genome assembly was polished using Racon version 1.3.3 (Vaser et al. 2017) and two rounds of Medaka version 1.0.1 (Oxford Nanopore Technologies 2018). For the 26,883,622 Illumina reads, adaptors, low-quality reads, and ambiguous nucleotides were removed from the generated short reads in CLC Genomics Workbench version 20 using very strict quality criteria with a quality limit of 0.001 and reads lower than 100 nt after trimming were discarded. The reads were mapped to the genome assembly and variants were detected using Basic Variant Detection in CLC Genomics Workbench version 20 using an expected ploidy of 1, ignoring variant positions with coverage lower than 10 and higher than 1,000,000 as well as non-specific read matches. The final hybrid genome assembly was then created by calling a consensus sequence in CLC Genomics Workbench 20 using majority vote. The completeness of the hybrid genome assembly was evaluated by Benchmarking Universal Single-Copy orthologs (BUSCO) version 5.0.0 (Seppey et al. 2019) analyses using the Ascomycota version 10 gene set.

The ITS region from the genome was obtained by using BLASTn in CLC algorithm in CLC Genomics Workbench version 20, and the ITS sequence from *Apiospora arundinis* CBS 106.12 (KF144883.1) as query. A multiple alignment was created between the ITS region of the isolate and 61 ITS regions from different *Apiospora* and *Arthrinium* species and one *Fusarium* species extracted from NCBI (Additional file 1, Table S1) using MAFFT (Rozewicki et al. 2019). A phylogenic tree was constructed using IQ-TREE (Nguyen et al. 2015) and a bootstrapping of 1,000. ModelFinder was used to find the best model for the tree construction (Kalyaanamoorthy et al. 2017). The tree was visualized using iTOL tree (Letunic and Bork 2021).

### RNA extraction and sequencing

*A. arundinis* AAU 773 was grown on glass beads (3 mm) submerged in liquid media, as described in Droce et al. 2013 (Droce et al. 2013). All the cultures were grown at 25 °C but on different media, with different illuminations and for different days (Additional file 1, Table S2). The cultures were cut in pieces, separated from the glass beads, and immediately frozen in liquid nitrogen. The samples were lyophilized overnight. RNA was extracted from the lyophilized mycelium with Qiagen RNeasy plant mini kit according to manufacturer’s protocol, except for the homogenization step, which was done by bead beating on a Precellys 24 homogenizer at 6,500 rpm for 30 s after adding 15 Precellys 03961VK05 beads (0.5 mm) and 450 *μ*L RLT buffer to each tube. The quality and concentration of the RNA was determined on NanoDrop One spectrophotometer and on Invitrogen Qubit Fluorometer using the HS assay kit according to manufacturer’s protocol. The fragment length was determined with Agilent 2200 Tapestation according to manufacturer’s protocol, using both RNA screentape and high sensitivity RNA screentape. RNA samples were prepared for sequencing using Illumina TruSeq Stranded RNA kit following manufacturer’s protocol. A sample of 10 *μ*M total DNA was sequenced by Quick Biology Inc. (California, USA) on an Illumina HiSeq 4000 sequencer generating 150 bp paired-end reads.

### Annotation and repeats in the genome

Repeats in the hybrid genome assembly were identified using RepeatMasker version 4.1.2 (Tarailo-Graovac and Chen 2009) using eukaryote as species. Barrnap version 0.9 (Seemann 2018) and tRNA-scan version 2.0.5 (Chan and Lowe 2019) were used to predict noncoding rRNA and tRNA genes. The hybrid genome assembly was annotated by using the RNA-seq reads from all the conditions (Additional file 1, Table S2). The reads were trimmed with Trim Galore version 0.6.4 (Krueger et al. 2021) using Cutadapt version 2.8 (Martin 2011). The trimmed reads were used to annotate the hybrid genome assembly using FunGAP version 1.1.0 (Min et al. 2017). The genes predicted using FunGAP were assigned functional annotation by blasting them against different databases including the non-redundant database of NCBI, InterProScan, EuKaryotic Orthologous Groups (KOG) using BLASTP (Additional file 1, Table S4). Only blast results under 10^-5^ e-value were included. Gene Ontology (GO) terms were assigned to the genes using Blast2GO (Götz et al. 2008). To analyze the content of carbohydrate-active enzymes (CAZymes), the predicted genes were submitted to the dbCAN2 meta server (Zhang et al. 2018) using HMMER. Only hits with an e-value under 10^-15^ and a coverage above 0.35 were included in the analysis (Additional file 1, Table S4). Secondary metabolite gene clusters were predicted using AntiSMASH version 5 (Blin et al. 2019) (Additional file 1, Table S4). Polyketide synthases (PKSs) was identified as highly reducing if congaing β-ketoreductase (KR), enoyl reductase (ER) and dehydrogenase (DH) domains, where they was identified as reducing if they only contained 1 or 2 of these domains. Non-reducing PKSs was identified as not containing any of these domains but a product template (PT) domain instead. A circular representation of the genome and the genomic features was visualized using Circos (Krzywinski et al. 2009).

### Gene expression, differential analysis, and enrichment analyses

The raw RNA-seq reads were trimmed using the CLC Genomics Workbench 20 to a quality of 0.05 and reads smaller than 15 bp after trimming were discarded. The trimmed reads were mapped to the annotated genome in CLC Genomics Workbench 20 using default parameters, and an expression matrix containing the total read count was calculated and extracted from the CLC Genomics Workbench 20. Differentially expressed genes (DEGs) were calculated using R version 4.1.1 (R Core 2021) in RStudio version 2022.7.1 (Team 2020) using the DESeq2 package (Love et al. 2014), with the following parameters: log2 fold changes ≥ 2 or ≤ -2, false discovery rate (FDR) ≤ 0.05. A principal component analysis (PCA) was constructed based on the rlog normalized expression matrix. A hierarchical clustering heatmap was generated for the calculated DEGs based on a z-score calculated from the rlog normalized expression matrix. Fisher’s exact test (FDR = 0.01) in BLAST2GO (Götz et al. 2008) was used to conduct the enrichment analyses. Average numbers of expressed genes (>10 expression level based on DESeq2 normalizing) associated with different CAZymes from the fungi cultivated on different media were determined and a z-score was calculated for each CAZymes. The results were visualized using R version 4.1.1 (R Core 2021) in RStudio version 2022.7.1 (Team 2020) using the ggplot2 package (Wickham 2016).

### Metabolomic analysis

*A. arundinis* AAU 773 was grown on glass beads submerged in liquid media. All the cultures were incubated at 25 °C (Additional file 1, Table S2). The cultures were frozen in liquid nitrogen and lyophilized overnight. The samples were homogenized by bead beating (0.25 to 0.5 mm) for 2x40 sec, with a 10 sec break. The samples were transferred to a glass tube covered with EtOAc:CH_2_Cl_2_:MeOH (3:2:1) 1% formic acid. Subsequently, the samples were sonicated for 30 min. and the solvent was afterwards decanted into new tubes and dried under a flow of N_2_ gas. The samples were dissolved in 200 uL methanol and analyzed by ultra-performance liquid chromatography-high resolution mass spectrometry (UHPLC-HRMS) (1290 UHPLC system armed with Agilent Poroshell 120 phenyl-hexyl column Agilent Technologies, Santa Clara, CA, USA). An Agilent 6545 quadrupole time of flight (QTOF) mass spectrometer with an Agilent Dual Jet Stream electrospray ionization source, run in positive mode was used for detection. The mass spectrometry method is further described by Subko et al. (Subko et al. 2021).

Raw datafiles from UHPLC-HRMS analysis were converted from Agilent ".d" format to the open source ".mzML" data format with MSConvert from ProteoWizard (version 3.0.19322-96421c9ae) (Chambers et al. 2012). Peak picking and data pre-processing was done with MZMine 3.0.21-beta (Pluskal et al. 2010) using the processing wizard function: instrument type was set to TOF and positive ionization mode was chosen. MS1 and MS2 noise levels were set to 1000 and 100, respectively, and minimum feature height was set to 10,000. Scan to scan m/z tolerance, feature to feature m/z tolerance, and sample to sample m/z tolerance were all set to 10 ppm. The HPLC system was set to UHPLC, and stable ionization across samples was ticked "on". Maximal peaks in chromatogram were set to 10. The minimum number of samples per aligned feature was set to two, and the minimum number of data points was set to three. Approximate feature FWHM was set to 0.2, intra-sample RT tolerance was set to 0.1, and inter-sample RT tolerance was set to 0.15. The function "only keep features with 13C" was ticked "on".

The Feature-Based Molecular Networking (FBMN) workflow (Nothias et al. 2020) from GNPS was used for molecular analysis. MS/MS fragment ions within 17 Da of the precursor ion were filtered and the top six fragment ions were window filtered with a 50 Da threshold. The tolerance for the precursor ion was 0.02 Da, and MS/MS fragment ion tolerance were set to 0.02 Da. The network was filtered with a cosine score threshold above 0.7 and at least five matching peaks. Edges between two nodes were kept if the respective nodes appeared as its other respective top 10 most similar nodes. The maximal size of a given molecular family was set to 100. MS/MS spectral search towards the GNPS spectral library (Horai et al. 2010; Wang et al. 2016) was also performed with a cosine score tolerance of 0.7 and at least five matched peaks.

### Statistical analysis of metabolomic data

Missing values were replaced by LoDs, normalized using sum, and scaled using Pareto scaling in MetaboAnalyst version 5.0 (Pang et al. 2021). A PCA plot based on log transformed normalized and scaled data was produced. The differential expression of metabolites was calculated in MetaboAnalyst version 5.0 (Pang et al. 2021) with the following parameters: log2 fold changes ≥ 1 or ≤ -1, false discovery ranges (FDR) ≤ 0.05. The feature-based molecular network was visualized using Cytoscape (Shannon et al. 2003).

### Gene synteny analysis

Candidate gene cluster for the identified compounds (ferricrocin, norlichexanthone and alkylcitric acids) was identified in the genome using the BLASTp algorithm in CLC Genomics Workbench version 20. Following protein sequences was used as query; NPS2 from *Fusarium pseudograminearum* CS3096 (XP_009260675.1) (Tobiasen et al. 2007), GsfA from *Penicillium aethiopicum* IBT 5753 (ADI24953.1) (Cacho et al. 2013) and NRRL3_11763 (akcA) from *Aspergillus niger* NRRL3 (Palys et al. 2020). Easyfig version 2.2.5 (Sullivan et al. 2011) was used to generate a gene synteny plot between the indentified candidate gene clusters and the similar gene clusters from *Fusarium pseudograminearum* CS3096, *Penicillium aethiopicum* IBT 5753 and *Aspergillus niger* NRRL3.

### Data availability

The generated genome assembly, the raw reads, and the RNA-seq reads for *A. arundinis* AAU 773 are available at the National Center for Biotechnology Information (NCBI) under the BioProject PRJNA891674. The genome assembly is deposited under the accession number JAPCWZ000000000 and the raw fastq files are deposited to the SRA database under accession number SRR23048519. The raw RNA-seq reads are deposited in the SRA database (See Additional file 1, Table S3 for accession numbers). The feature-based molecular network can be found through the GNPS website using the following link: https://gnps.ucsd.edu/ProteoSAFe/status.jsp?task=617431dfb9e041549738638f5f4748f6. The mass spectrometry data are available at the following link: ftp://MSV000090801@massive.ucsd.edu, MassIVE ID: MSV000090801.

## Results and discussion

### Sequencing and de novo assembly of the genome

**Fig. 1:**
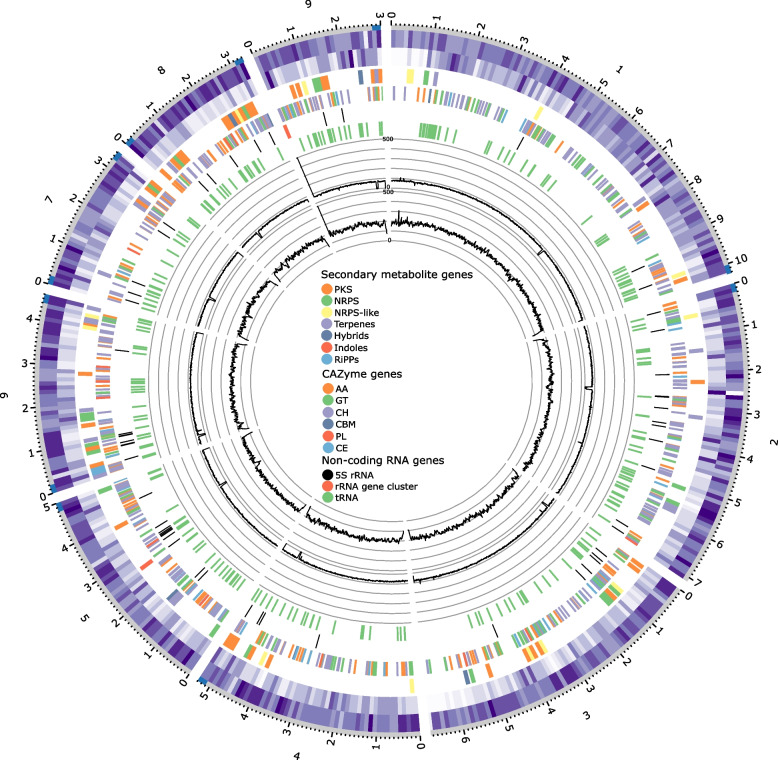
A circular representation of the genome of *A. arundinis* AAU 773. Gene density and repeat density were calculated using bins of 10,000 kbp. Circles from the outside to the inside show the position of the telomeric regions, the gene density, the repeat density, the placement of the secondary metabolite genes, the placement of the CAZyme genes, the position of the rRNA genes, the placement of the tRNA genes, coverages of Illumina reads, and coverages of Nanopore reads

The hybrid genome assembly (based on both long Oxford Nanopore Technology (ONT) reads and short Illumina reads) consisted of 48.8 Mb with a GC content of 52.1% (Table 1) comparable to previously sequenced genomes of this genus (Li et al. 2020; Heo et al. 2021; Majeedano et al. 2022; Petersen et al. 2022). The genome was assembled by using 9.948 Gb from 257,189 long reads and 11.3 Gb from 18,781,708 short reads. Polishing of the genome assembly using short reads corrected a total of 7,974 bases. This corresponds to an estimated per base accuracy of the initial long-read genome assembly of 99.984% assuming that the accuracy of the hybrid genome assembly was 100%. The genome assembly comprised 10 contigs, of which one was identified as a mitochondrial genome, similar (99.6%) to a mitochondrial genome from another *A. arundinis* isolate (KY775582). A highly contiguous genome assembly was also evident from a N50 of 5.3 Mb and a N99 of 3.0 Mb. Three contigs were observed to have telomeric regions on both ends (Fig. 1), indicating that they represent complete chromosomes. Four contigs were noted to have telomeric regions on only one end, and only one contig had non-telomeric regions. The Benchmarking Universal Single-Copy Orthologs (BUSCO) analyses showed a BUSCO completeness of 99.4%, which demonstrated a highly complete genome assembly.

**Table 1:** Summary of genome statistic

**Genome characteristics**		
Assembly	Genome assembly size (Mb)	48.8
	Nanopore coverage (x)	202
	Illumina coverage (x)	231
	Number of contigs	10
	Longest contig (Mb)	10.2
	N50 (Mb)	5.3
	N99 (Mb)	3.0
	GC (%)	52.1
BUSCO	Complete BUSCOs (%)	99.4
	Complete and single-copy BUSCOs (%)	98.9
	Complete and duplicated BUSCOs (%)	0.5
	Fragmented BUSCOs (%)	0.5
	Missing BUSCOs (%)	0.1
	Total number for BUSCOs (n)	1315

### Phylogenic classification of the isolate

**Fig. 2:**
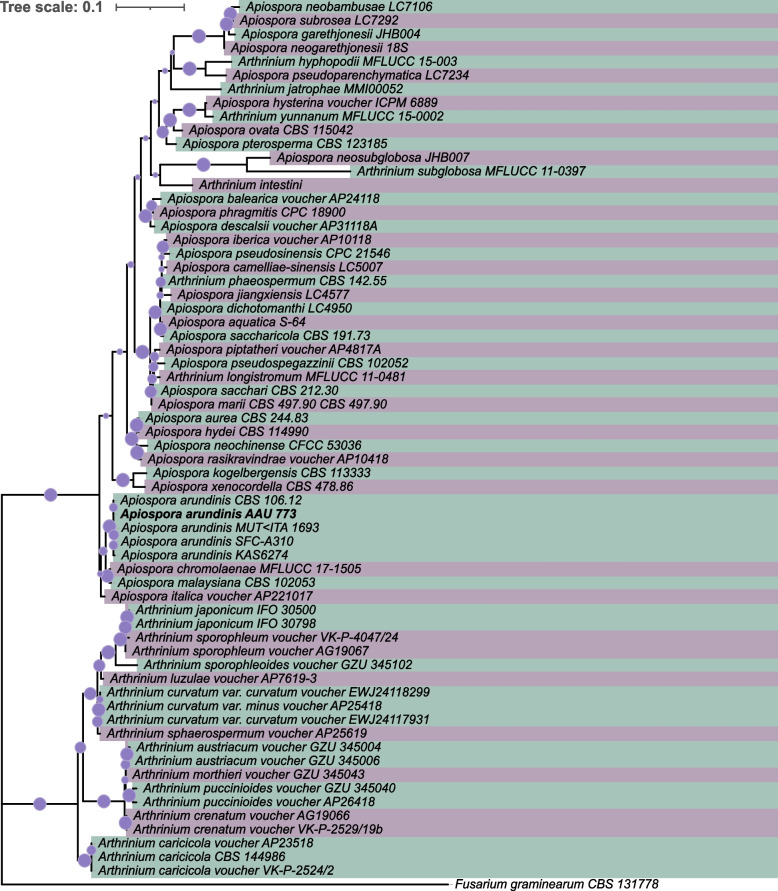
Phylogenic tree of *Apiospora* and *Arthrinium* based on sequences of ITS regions. Dots on branches represent bootstrap values (1,000). The tree was rooted with the ITS region from *Fusarium graminearum* (CBS:131778)

The phylogenic position of the isolated fungus was investigated by a phylogenetic analysis based on ITS regions from previously classified species (Additional file 1, Table S1, Fig. 2). The phylogenetic tree was divided into two overall branches, one consisting of *Apiospora* species and the other of *Arthrinium* species. The topology of the resulting tree was consistent with the findings observed by others (Crous et al. 2021; Pintos and Alvarado 2021; Tian et al. 2021), which showed a delimitation of these two groups. The isolated fungus was identified as belonging to the *Apiospora* genus and was found to be closely related to *A. arundinis*, which formed a phylogenetic clade, implying that it is also an *A. arundinis* species, and was thus named *Apiospora arundinis* AAU 773.

### Genomic features of *A. arundinis* AAU 773

#### Repeat sequences and non-coding RNA genes in the genome

The length of the interspersed repeat of the *A. arundinis* AAU 773 genome was determined to be 976 kbp, covering a total of 2% in the genome (Fig. 1). Thereof, the short interspersed nuclear elements (SINEs) and the long interspersed nuclear elements (LINEs) accounted for 0.09% and 0.15%, respectively. Long terminal repeat (LTR) elements were observed to account for 1.08%, DNA transposons for 0.51% and 0.18% was observed to be unclassified repeats. The tandem repeat length in the *A. arundinis* AAU 773 genome was observed to be 581 kbp, covering a total of 1.2% of the genome.

A total of 356 non-coding tRNA and rRNA genes were predicted in the genome of *A. arundinis* AAU 773 (Fig. 1). Of these genes, 277 were predicted to be tRNA genes, whereas 13 were predicted to be pseudogenes. We identified 66 rRNA genes in the genome, of which 56 were predicted to be 5S rRNA genes. One rRNA gene cluster, containing four 5.8S rRNA genes, three 18S rRNA genes, and three 28S rRNA genes, was observed to be located at the end of contig 9 (Fig. 1). The coverage of both the Illumina reads and the Nanopore reads were, however, high in this region compared to the average converges (Fig. 1). This indicates that the true number of these rRNA genes is in fact higher than observed here.

#### Gene prediction and functional annotation of the genome

We investigated the genomic potential of the fungus by predicting the gene structure in the genome. A total of 15,725 predicted protein-coding genes was obtained. The average open reading frame length was 1,407 bp and the average protein size was 469 amino acids..

The predicted genes were functionally annotated using different databases. It was possible to functionally annotate 13,619 of the predicted genes using the non-redundant database of NCBI, comprising 86.6% of the total number of predicted genes. Functional annotation using InterProScan yielded a total of 13,194 functionally annotated genes, comprising 83.9% of the total number of predicted genes. The predicted genes were also functionally annotated using GO, of which 10,845 predicted genes could be assigned to at least one GO term. The fundamental GO terms: biological process, molecular function, and cellular component, were assigned to 5,175, 7,442, and 4,216 genes, respectively. Functional annotation of the predicted genes using the KOG database yielded a total of 6,190 genes that were assigned to at least one KOG category. The largest group was observed to be "general functional prediction only" followed by "posttranslational modification, protein turnover, chaperones", "secondary metabolite biosynthesis, transport, and catabolism", and "lipid transport, and metabolism" (Additional file 1, Table S4).

*Apiospora arundinis* has been isolated from different plants (Martínez-Cano et al. 1992; Bagherabadi et al. 2014; Chen et al. 2014; Thangaraj et al. 2019; Liao et al. 2022), suggesting it has the potential to produce high numbers of enzymes able to degrade complex carbohydrates, since these are major constituents of plant material. This also indicates that they are generalists rather than specialists. The predicted genes were assessed for genes that could be assigned to a CAZyme class. A total of 692 genes fulfilled the criteria and could be assigned to the dbCAN2 database (Fig. 1 and Additional file 1, Table S4). In total, the fungi features 317 glycoside hydrolases (GHs), 196 auxiliary actives (AA), 102 glycosyltransferases (GTs), 77 carbohydrate esterases (CEs), 12 polysaccharide lyases (PLs), and 10 carbohydrate-binding modules (CBMs) (Additional file 1, Table S4). This shows that *A. arundinis* AAU 773 are able to produce a high number of different CAZymes, as seen for other pathogenic fungi (Zhao et al. 2014). A high number of different CAZymes is also often observed in generalists, as generalists can utilize a lot of different substrates (Zhao et al. 2014). Therefore, the comprehensive gene diversity supports the suggestion that *Apiospora* are generalistic fungi.

The biosynthetic potential for production of secondary metabolites was also investigated. This analysis demonstrated that this genome has a high biosynthetic potential for production of secondary metabolites (Fig. 1). A total of 106 secondary metabolite genes were predicted in the genome, of which a variety of different classifications were assigned to the genes: polyketide synthases (PKSs) (43), nonribosomal peptide synthetases (NRPSs) (19), terpenes (19), PKS-NRPS hybrids (3), NRPS-PKS hybrids (2), indoles (1), and post-translationally modified peptides (RiPPs) (2) (Additional file 1, Table S4). Significantly, a majority of these predicted secondary metabolite clusters genes had functions that remained unknown, highlighting the potential of exploring *Apiospora* for the discovery of novel secondary metabolites. The genome of *A. arundinis* AAU 773 is relatively rich in gene clusters associated with PKSs compared to other filamentous fungi, which are popular choices for mining genomes for secondary metabolite gene clusters, such as *Fusarium* and *Aspergillus* that usually contain 10-40 PKSs (Lin et al. 2012; Hansen et al. 2015; Brown and Proctor 2016). Of the 43 PKS genes that are observed in the genome of *A. arundinis* AAU 773, 25 can be classified as highly reducing PKSs while 12 are partially reducing PKSs and seven are non-reducing PKSs. Additionally, 5 genes were identified that possessed both a PKS and NRPS domain, with these domains located either at the N-terminal or the C-terminal of the resulting enzymes. Comparing the AntiSMASH results for the secondary metabolite clusters containing PKS associated genes, with known secondary metabolites produced by *Arthrinium* and *Apiospora* (Overgaard et al. 2022), it is likely that the fungus can produce 6-MSA derived metabolites, Depudecin-like compounds, Alternapyrones, Asperthecin, Arthpyrone, and lipopeptides. This however must be evaluated further to fully confirm.

#### Transcriptomic analysis of *A. arundinis* AAU 773

A transcriptomic analysis using RNA-sequencing was carried out to examine the gene expression response of *A. arundinis* AAU 773 cultivated on various media types. Hay trace solution medium (HTM), yeast extract medium (YES), penicillin production medium (PPB), ammonium (dihydrogen) phosphate medium (APM), potato dextrose broth (PDB), and brown rice medium (BRM) were used to cultivate the fungus. The HTM and YES media contain either complex carbohydrates or simple carbohydrates (starch), respectively. We therefore expected that genes associated with enzymes capable of degrading complex carbohydrates would be overexpressed when the fungi were cultivated on HTM and only a few genes would be expressed when the fungi were cultivated on YES.

**Fig. 3:**
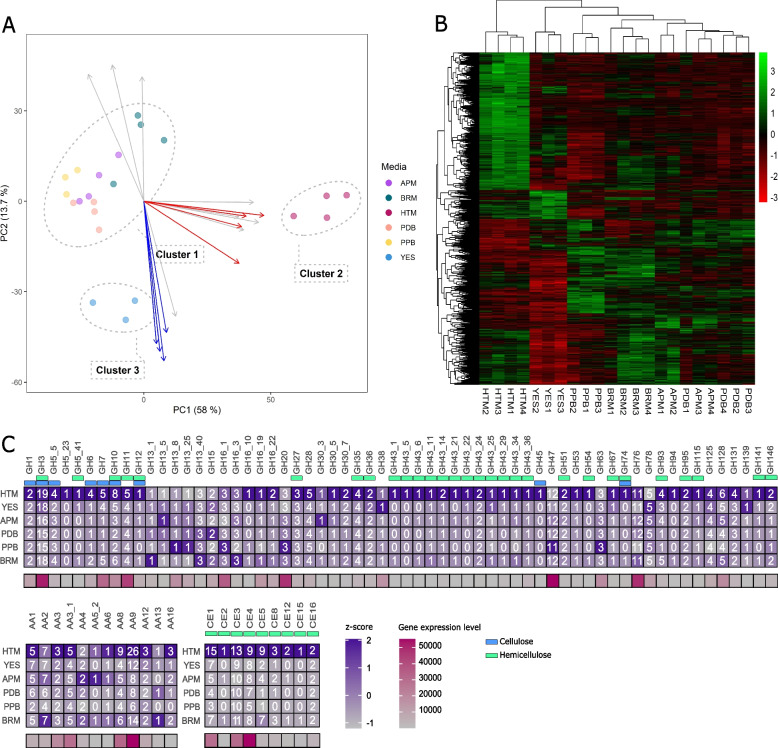
Transcriptomic analysis of *A. arundinis* AAU 773 cultivated on different media. **A** Principal component analysis of the gene expression in fungus cultivated on different media. Arrows represent the eight highest loadings of PC1 and PC2. Red arrows represent genes associated with CAZymes and blue arrows represent genes with GO terms associated with transport. **B** Hierarchal clustering heatmap of upregulated and downregulated DEGs. Colors show the z-score of the transformed gene expression. C. CAZymes observed to be expressed by the fungus cultivated on different media. Only CAZymes with at least one z-score above 1.6 were included. The numbers represent the amount of CAZymes expressed by the fungus cultivated on this medium. Lower panel presents the sum of gene expression level observed in all samples

Based on the gene expression dataset, an unsupervised principal component analysis (PCA) was carried out to investigate the dissimilarity between the samples (Fig. 3A). The PCA model revealed a low variance between the replicates, indicating a consistent and reliable result from the transcriptomic analysis. Additionally, the model identified three distinct patterns of gene expression. The gene expression levels of the fungus grown on APM, PDB, PPB, and BRM (cluster 1) were similar, and cultivating the fungus on these various media does not appear to have a significant impact on the gene expression levels. Similarity between these samples was also supported by the hierarchical clustering (Fig. 3B). The PCA model, however, showed a more distinct gene expression level when the fungus was cultivated on HTM (cluster 2) and YES (cluster 3) from cluster 1. Cluster 2 was clearly separated from cluster 1 along PC1 (58%), indicating a higher change in gene expression levels when the fungus was cultivated on YES than when the fungus was cultivated on HTM. In contrast, cluster 3 was only separated from cluster 1 along PC2 (13.7%), showing a more similar gene expression level between cluster 3 and cluster 1 than between cluster 2 and cluster 1. Cluster 3 was correlated with several genes that could be associated with GO terms associated with transport (Fig. 3A). This indicated that the fungus expressed a high level of genes related to transport when it was cultivated on YES. Cluster 2 appeared to be correlated with multiple CAZyme-related genes (Fig. 3A). This suggested that when the fungus was cultivated on HTM, it expressed a high level of these types of genes (Fig. 3A).

We wanted to further investigate the expression of CAZyme-related genes from the fungus cultivated on the different media (Fig. 3C). It was discovered that genes linked to CAZymes were highly expressed when the fungus was cultivated on HTM compared to the fungus in cluster 1 (Fig. 3C). A total of 86.6% of the CAZymes in the genome were expressed when the fungus was cultivated under these conditions. The highest number of expressed genes associated with a single CAZyme class was observed to be from the AA9 family, where a total of 26 out of 30 genes were expressed when the fungus was cultivated on HTM. This class mainly contains lytic polysaccharide monooxygenases (LPMOs). A high number of expressed LPMOs can provide the fungus with enzymes to degrade plant cell walls more (Morgenstern et al. 2014; Jagadeeswaran et al. 2021). Studies have shown that nearly all fungi that are able to degrade lignocellulose contain LPMO genes (Morgenstern et al. 2014; Johansen 2016; Jagadeeswaran et al. 2021), and that some pathogenic fungi that do not have the ability to produce cellulases, or only a small number of cellulases, still harbor LPMO genes (Pham et al. 2019). Fungi that are more aggressive when infecting plants also tend to have a high number of LPMO genes (Morgenstern et al. 2014; Jagadeeswaran et al. 2021). This shows that this CAZyme family is highly relevant and is important for pathogenic fungi and their ability to infect plants. Several different enzymes are required for total degradation of the cellulose in the plant cell wall (Sánchez 2009; Andlar et al. 2018). Endo-β-1,4-glucanases (EC 3.2.1.4) (mainly observed in GH5_5, GH6 and GH7) and exo-β-1,4-glucanases (EC 3.2.1.91) (mainly observed in GH6) are required for the breakdown of cellulose into smaller molecules such as cellobiose. Cellobiose can then further be cleaved into D-glucose by β-glucosidases (EC 3.2.1.21) (mainly observed in GH3). *A. arundinis* AAU 773 was observed to have the ability to produce a high amount of all these types of enzymes, giving it the ability to fully degrade cellulose (Fig. 3C, Additional file 1, Table S4). Degrading hemicellulose from plant cell walls is, however, a more complex reaction since the building blocks of hemicellulose are far more diverse than cellulose (Lange 2017). Here, a wide range of enzymes are required to target the different structures observed in hemicellulose. *A. arundinis* AAU 773 was observed to express a high range of different genes associated with degradation of hemicellulose when it was cultivated on HTM, hence giving it the ability to utilize these complex and diverse carbohydrates as well (Fig. 3C).

In general, a change in the transcriptome was observed in *A. arundinis* AAU 773 as a response to culturing on different growth media. Furthermore, a high expression of CAZyme-related genes could be observed when the fungus was cultivated on HTM. The ability of *A. arundinis* AAU 773 to modulate its gene expression in response to changes in growth media reflects its capacity to thrive in diverse ecological niches. Suggest a dynamic and adaptable nature of the fungus. Additionally, these findings contribute to our knowledge of fungal biology and enhance our understanding of how fungi respond to their environments.

#### Differential expression of genes

The difference in the gene expression of the fungus cultivated on different media was further explored (Additional file 1, Table S5 and S6). A total of 1,168 differentially expressed genes (DEGs) was observed between cluster 1 and cluster 2. A total of 762 DEGs were upregulated in cluster 2, where 405 DEGs were downregulated in cluster 2. This showed that a higher number of genes are expressed when the fungus was cultivated on HTM. When looking at the DE-analysis between cluster 1 and cluster 3, a total of 981 DEGs were observed, whereof 204 DEGs were observed to be upregulated and 777 DEGs were observed to be downregulated when the fungus was cultivated on YES. This showed that the fungus only expressed a relatively low number of genes when it was cultivated on YES compared to when it was cultivated on APM, PDB, PPB, and BRM.

### Enrichment analysis of functionally annotated DEGs

**Fig. 4:**
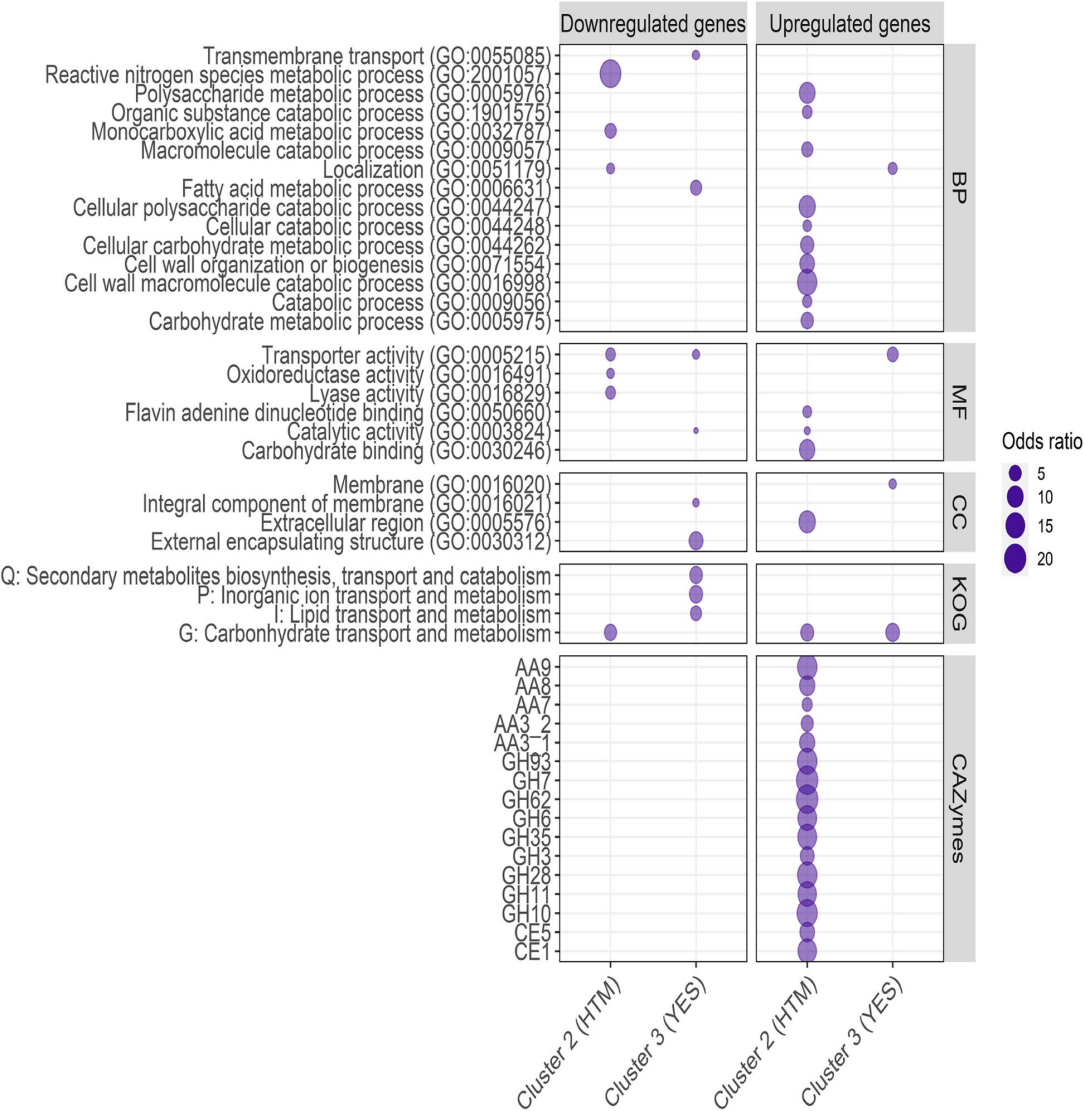
Enrichment analysis of DEGs calculated between the transcriptome of *A. arundinis* AAU 773 cultivated on different media as a response to growth media. Only significantly enriched terms were included (FDR < 0.01). The GO terms were reduced to a higher term if such a term was observed. For full data, see Additional file 2, Figure S1. BP: biological process, MF: molecular function, CC: cellular component

We also want to investigate which genes were responsible for the differences in the transcriptome of the fungus grown on different growth media (Fig. 4, Additional file 2, Figure S1) It was revealed that upregulated DEGs observed between cluster 1 and cluster 2 were enriched with the biological process GO terms “organic substance catabolic process” and “carbohydrate metabolic process” and several terms connected to these terms (Fig. 4). These upregulated DEGs were also enriched with the molecular function GO terms “catalytic activity” and “carbohydrate binding” and several terms connected to these terms (Fig. 4). A total of 16 CAZyme families were also observed to be enriched in these upregulated DEGs with 9, 4, and 2 of them observed to belong to glycoside hydrolases (GH), auxiliary activities (AA), and carbohydrate esterases (CE), respectively. Again, this shows that the fungus expresses a high number of genes associated with the degradation of complex carbohydrates when cultivated on HTM.

The enrichment test of the DEGs observed between cluster 1 and cluster 3 showed that the upregulated and downregulated DEGs were enriched with different GO terms associated with transport (Fig. 4, Additional file 2, Figure S1). The enrichment test of the KOG terms showed that cluster 3 was enriched with the term “Carbohydrate transport and metabolism”. Cluster 1 was enriched with the terms “Secondary metabolites biosynthesis, transport and catabolism”, “Lipid transport and metabolism", and “Inorganic ion transport and metabolism”. Upregulated DEGs in cluster 1 were also enriched with the molecular function GO term “Oxidoreductase activity” and several terms connected to this term. The DEGs in cluster 1 had in general, more enriched terms than the DEGs in cluster 3.

### Metabolomic analysis of *A. arundinis* AAU 773

**Fig. 5:**
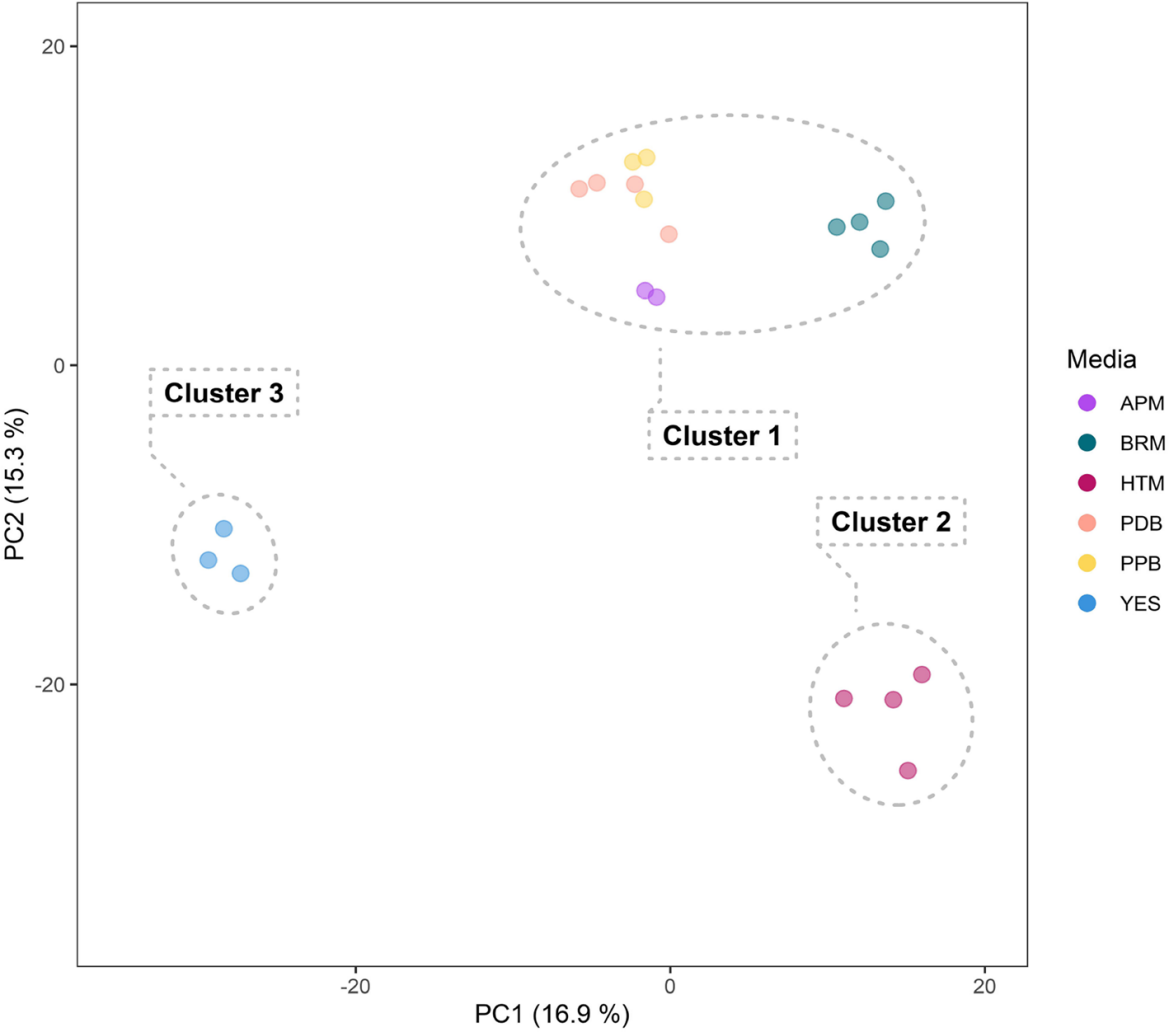
Principal component analysis of the *A. arundinis* AAU 773 metabolomes when cultivated on different media

The variability in the metabolome of *A. arundinis* AAU 773 cultivated on various media was also analyzed using a metabolomics approach (Additional file 1, Tables S7 and S8). The same media used for the transcriptomic analysis were used to cultivate the fungus for the metabolomic analysis. Based on the metabolomic data, an unsupervised PCA model was first created to investigate the dissimilarity in the metabolome depending on the media (Fig. 5). The model showed a clear clustering of samples that were cultivated on the same media, indicating a reliable representation of the dataset. As in the transcriptomic analysis, the metabolomes of the fungus when cultivated on APM, PDB, PPB, and BRM seemed to be similar. It was also observed that the metabolomes of the fungus when cultivated on YES and HTM were separated from the other metabolomes along PC1 (16.9%) and PC2 (15.3%), respectively.

### Feature-based molecular network

**Fig. 6:**
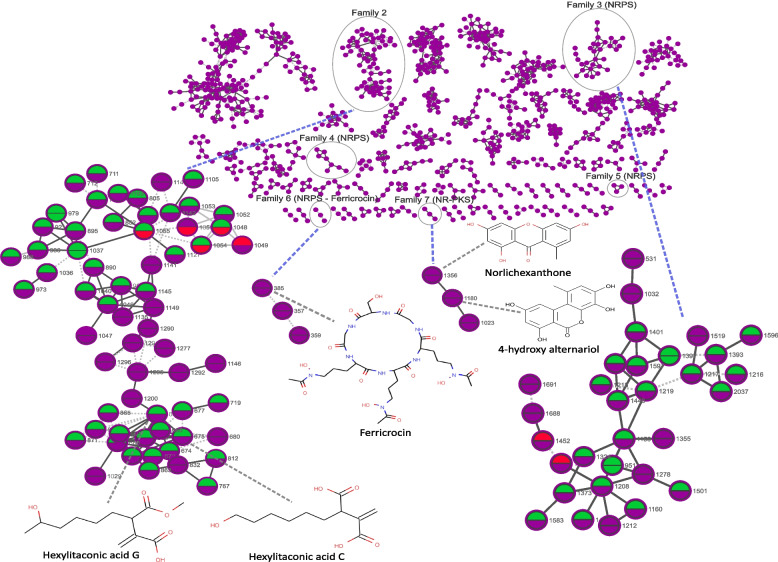
Feature-based molecular network of *A. arundinis* AAU 773 cultivated on different media. Four clusters are highlighted, ID of each node is presented on the side of the node. Color of lower circles in the nodes represent features that are overproduced (green) or underproduced (red) in the metabolomes of fungus cultivated on HTM. Color of upper circles in the nodes represent features that are overproduced (green) or underproduced (red) in the metabolomes of fungus cultivated on YES. Dotted edges represent MS1 feature shape correlation, straight lines represent MS2 similarity

The metabolic potential of *A. arundinis* AAU 773 cultivated on different media was explored using a feature-based molecular network based on the metabolomic data produced (Fig. 6). A total of 90 chemical families (>3 nodes) were observed from the grouping of the metabolite features.

**Fig. 7:**
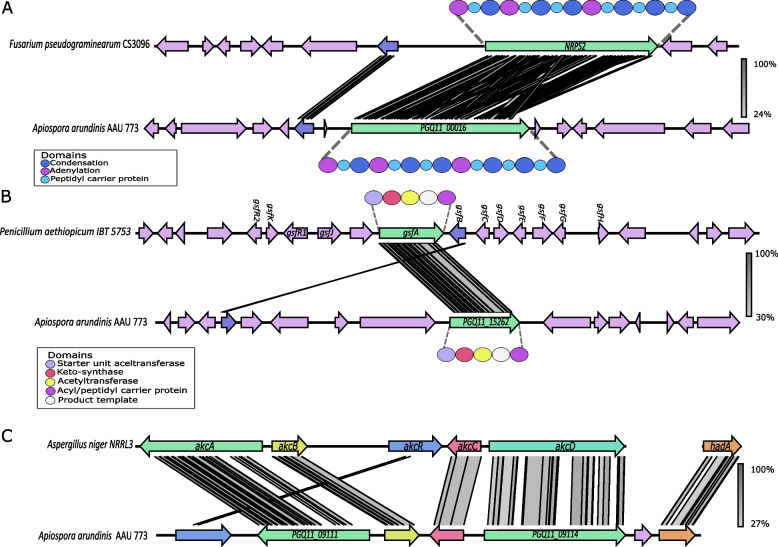
Gene synteny plot. **A** Synteny analysis between NRPS2 (ferricrocin) gene cluster in *Fusarium pseudograminearum* CS3096 (Tobiasen et al. 2007) and an NRPS gene cluster in *A. arundinis* AAU 773. **B** Synteny analysis between an NR-PKS (norlichexanthone) gene cluster in *Penicillium aethiopicum* IBT 5753 (Cacho et al. 2013) and an NR-PKS gene cluster in *A. arundinis* AAU 773. **C** Synteny analysis between a fatty acid synthases (alkylcitric acids) gene cluster in *Aspergillius niger* NRRL3 (Palys et al. 2020) and a fatty acid synthases gene cluster in *A. arundinis* AAU 773. Arrows represent the different genes in the gene clusters

The *Apiospora* genus has been shown to be capable of producing a high number of secondary metabolites (Overgaard et al. 2022). We therefore wanted to investigate which of the chemical families in the feature-base molecule network had possible secondary metabolites (Fig. 6 and Additional file 1, Table S9). Using an *in silico* approach it was possible to predict four chemical families as nonribosomal peptides (NRPs) families (Fig. 6). Chemical family 3 was predicted to be a family containing compounds produced by one or several different nonribosomal peptide synthetases (NRPSs). This chemical family contained several features with a high mass-to-charges (mz) ratio, indicating that its compounds are not only chemically alike but also had a high molecular weight. This *in silico* prediction of the features in chemical family 3 suggested several NRPS compounds with similar structure of either seven, six, or five amino acids. A high production of several features in this family was also observed, and only a few features were not produced on some media. This indicates that these compounds are important for the fungus regardless of what media it is cultivated on. Chemical family 6 was identified as a family only containing ferricrocin features, which in other studies have been shown to be produced by an NRPS gene cluster (Tobiasen et al. 2007). We identified a gene possessing an identical domain structure to the NRPS2 gene found in *Fusarium pseudograminearum*, a gene that has been demonstrated to be responsible for the biosynthesis of ferricrocin (Fig. 7A). This gene was also observed to have similarity (40.6%) to the NRPS2 gene found in *Fusarium pseudograminearum.* We also observed a high similarity (56.2%) between the gene encoding L-ornithine N5-oxygenase (*SIDA*) in *F. pseudograminearum* and a gene in *A. arundinis* AAU 773. However, other genes within the gene clusters in the two species showed no homology (Fig. 7A). Another chemical family was identified as harboring a compound, norlichexanthone. This compound was earlier observed in to be produced by other *Apiospora* species (Wang et al. 2014; Liao et al. 2018). Norlichexanthone has been shown to be produced by the non-reducing PKS (NR-PKS) *gsfA* gene in *Penicillium aethiopicum* (Cacho et al. 2013). It was possible to identify an NR-PKS gene with high similarity (76.5%) to the *gsfA* gene in the genome of *A. arundinis* AAU 773 (Fig. 7B). This gene has the same domain structure as the *gsfA* gene and could be responsible for the production of this compound *in A. arundinis* AAU 773 (Fig. 7B*)*. It was not, however, possible to find a similarity between other genes of this cluster besides an O-methylation gene (*gsfB*) which was observed to have a similarity on 32%.

Chemical family 2 was observed to have two annotated features: hexylitaconic acid C and hexylitaconic acid G; both related to alkylcitric acids (Fig. 6C). These compounds have been shown to be produced by a fatty acid gene cluster in *Aspergillus niger* (Palys et al. 2020). It has been shown that the genes *akcA*, a*kcB*, *akcC*, and *akcD* were the responsible backbone genes in the production of these compounds. The *akcR* was also found to be the transcription factor for this gene cluster. It was possible to identify a gene cluster in the genome of *A. arundinis* AAU 773 with genes similar to these backbone genes (Fig. 6C). Furthermore, a transcription factor was observed in the same gene cluster (*akcR*), but with a lower similarity (27%) to the *akcR* gene. Palys et al. (2020) identified a hexylaconitic acid decarboxylase gene (*hadA*) that was also responsible for the formation of hexylitaconic acid B, a compound that hexylitaconic acid C and hexylitaconic acid G are produced from. Interestingly, they did not observe this gene to be co-localized with the other genes. Here, we identified this gene in the same gene cluster as the other genes.

In summary, the metabolomic analyses of *A. arundinis* AAU 773 unveil a fungus cable of producing an array of diverse compounds. These findings contribute valuable insights into the intricate metabolic capabilities of *A. arundinis* AAU 773 across different cultivation media, paving the way for future investigations into the functional roles and ecological significance of these identified compounds.

### Differential expression of metabolites

We also wanted to investigate which features differentiated the metabolomes of *A. arundinis* AAU 773 cultivated on YES and HTM from the other metabolomes (Fig. 6, Additional file 1, Tables S10 and S11). Notably, when the fungus was cultivated on HTM, a total of 312 features were observed to be overproduced, while 55 features were underproduced. This suggests a distinctive metabolic response of *A. arundinis* AAU 773 to the HTM medium, indicating potential biochemical pathways or processes influenced by this specific cultivation media. Similarly, cultivating the fungus on YES revealed significant differences, with a total of 615 and 36 features observed to be overproduced and underproduced, respectively. The identification of these overproduced and underproduced features reveal a metabolic change in *A. arundinis* AAU 773 as response to different growth environments.

The chemical family 3, which was observed to contain several features probably produced by one or several NRPSs, was observed to harbor several features that were mostly overproduced on HTM and YES (Fig. 6). The metabolome of the fungus when cultivated on YES was also observed to overproduce certain chemical family 2 features, including hexylitaconic acid C and hexylitaconic G (Fig. 6).

## Conclusion

We have successfully generated a highly comprehensive and contiguous genome assembly of the isolated fungus *Apiospora arundinis* AAU 773. Based on functional predictions of protein-coding genes, this fungus appears to have the capacity to produce a wide array of secondary metabolites and carbohydrate-active enzymes (CAZymes). Notably, several of these secondary metabolite gene clusters were previously unknown, rendering this fungus exceptionally intriguing for the exploration and discovery of novel compounds and valuable enzymes. When cultivated on different growth media, namely HTM and YES in contrast to PPB, PDB, APM, and BRM, we observed significant variations in the gene expression patterns and metabolomic profiles of the fungus. When cultivated in HTM, the fungus exhibited heightened expression of numerous genes associated with carbohydrate metabolism. Conversely, when grown in YES, the fungus displayed a relatively modest expression of genes. Furthermore, our metabolomic analysis unveiled the fungus's ability to produce four known compounds: ferricrocin, norlichexanthone, and two alkylcitric acids. We were able to pinpoint the likely gene clusters responsible for the synthesis of these compounds.

### Supplementary Information

**Additional file 1: Table S1-S11**.

**Additional file 2: Figure S1.** Full enrichment analysis of DEGs calculated between the transcriptome of *A. arundinis* AAU 773 cultivated on different media as a response to growth media.

## Data Availability

The generated genome assembly, the raw reads, and the RNA-seq reads for *A. arundinis* AAU 773 are available at the National Center for Biotechnology Information (NCBI) under the BioProject PRJNA891674. The genome assembly is deposited under the accession number JAPCWZ000000000 and the raw fastq files are deposited to the SRA database under accession number SRR23048519. The raw RNA-seq reads are deposited in the SRA database (See Additional file 1, Table S3 for accession numbers). The feature-based molecular network can be found through the GNPS website using the following link: https://gnps.ucsd.edu/ProteoSAFe/status.jsp?task=617431dfb9e041549738638f5f4748f6. The mass spectrometry data are available at the following link: ftp://MSV000090801@massive.ucsd.edu, MassIVE ID: MSV000090801.
