# Supplementary material for: Apiospora arundinis, a panoply of carbohydrate-active enzymes and secondary metabolites
Source: IMA Fungus. 2024 Apr 7;15:10. doi: 10.1186/s43008-024-00141-0 (PMC10999098; doi:10.1186/s43008-024-00141-0)
Supplement: Supplementary file 2 — Additional file 2: Figure S1. Full enrichment analysis of DEGs calculated between the transcriptome of A. arundinis AAU 773 cultivated on different media as a response to growth media. [file 43008_2024_141_MOESM2_ESM.docx]

Additional file 2



Figure S1 – Full enrichment analysis of DEGs calculated between the transcriptome of A. arundinis AAU 773 cultivated on different media as a response to growth conditions. Only significantly enriched terms were included (FDR < 0.01). The GO terms were reduced to a higher term if such a term was observed. BP: biological process, MF: molecular function, CC: cellular component.
